# Bibliometric analysis of metformin as an immunomodulator (2013–2024)

**DOI:** 10.3389/fimmu.2024.1526481

**Published:** 2025-01-08

**Authors:** Tongyi Zhou, Yunfeng Yu, Liu Li, Xiu Liu, Qin Xiang, Rong Yu

**Affiliations:** ^1^ School of Traditional Chinese Medicine, Hunan University of Chinese Medicine, Changsha, China; ^2^ Hunan Key Laboratory of Traditional Chinese Medicine Prescription and Syndromes Translational Medicine, Hunan University of Chinese Medicine, Changsha, China

**Keywords:** metformin, immunomodulator, tumor immune microenvironment, immune checkpoint inhibition, aging, inflammation, bibliometric analysis

## Abstract

**Background:**

Metformin, the frontline treatment for diabetes, has considerable potential as an immunomodulator; however, detailed bibliometric analyses on this subject are limited.

**Methods:**

This study extracted 640 relevant articles from the Web of Science (WOS) Core Collection and conducted visual analyses using Microsoft Excel, VOSviewer, and CiteSpace.

**Results:**

The findings showed that research on the immunomodulatory function of metformin has grown steadily since 2017, with China and the United States being the leading contributors. These studies have mostly been published in journals such as *the International Journal of Molecular Sciences, Cancers, Frontiers in Immunology, and Scientific Reports*. Keyword co-occurrence analysis highlighted metformin’s role as an immunomodulator, particularly in the context of the tumor immune microenvironment, immunosuppressive checkpoints, and metformin derivatives. Recent research has highlighted metformin’s application in aging, autoimmune diseases, COVID-19, and tuberculosis. Additionally, its role in regulating inflammation and gut microbiota is also being investigated.

**Conclusion:**

Overall, the immunomodulatory effects of metformin were investigated in anti-tumor, antiviral, anti-aging, and autoimmune disease research. This highlights the scope of metformin use in these fields, while also significantly enhancing its clinical value as a repurposed drug.

## 1 Introduction

Metformin, a guanidine derivative of herbal goat bean, has long been the primary oral hypoglycemic agent used to treat type 2 diabetes (T2DM). It is recognized for its high-efficiency hypoglycemic and cardiovascular protective effects, without increasing body weight or the risk of hypoglycemia (1). This drug has garnered significant attention due to its extensive clinical application and potential health benefits. Preclinical and observational studies have demonstrated the promising potential of metformin in treating conditions such as obesity, metabolic syndrome, osteoporosis, rheumatoid arthritis, aging, periodontitis, cancer, liver disease, kidney disease, inflammatory bowel disease, tuberculosis, coronavirus disease 2019 (COVID-19), osteoarthritis, and other autoimmune inflammatory rheumatic diseases (2–4). However, the specific mechanisms and clinical efficacy of these effects requires further investigation.

Over the past decade, research has displayed that in addition to its hypoglycemic effects, metformin can regulate cell energy metabolism, proliferation, growth, inflammation, endoplasmic reticulum stress (ERS), and autophagy, as well as improve intestinal flora (5–8). Mounting evidence shows that metformin is an effective immune system activator (9). It not only regulates the host immune function but also demonstrates an enhanced therapeutic efficacy in treating the aforementioned diseases and overcomes immunotherapy resistance, although the molecular mechanisms of these effects are not completely understood (10, 11). The unexpected immunomodulatory benefits of metformin were initially identified by Pearceet al. (12), who demonstrated that metformin enhances the generation and persistence of memory CD8+ T cells by activating fatty acid oxidation in T cells, revealing a novel mechanism for improving immune function. This finding was later supported by Finisguerra et al. (13). Many experimental and clinical studies have demonstrated that metformin can regulate tumor-infiltrating effector immune cells and inhibit immunosuppressive cells in various tumor models of breast, liver, lung, head and neck, and colorectal cancer (14–18). Metformin also exhibits an anti-tumor effect by regulating programmed death-1(PD-1)/programmed death-ligand 1(PD-L1) immunosuppressive checkpoints, which may involve ERS and adenosine monophosphate-activated protein kinase (AMPK) pathways (19, 20). Other studies have shown that metformin prevents age-related ovarian fibrosis by balancing the number of fibroblasts, myofibroblasts and immune cells (21). Furthermore, metformin exerts its anti-inflammatory and immunosuppressive effects by regulating the macrophage expression of the plasticity factor, zinc finger e-box binding homeobox 1(ZEB1) (22). However, in cases of obesity (23), atherosclerosis (24), and bone-related lesions (25), metformin induces M2 polarization. Rodriguez’s team found that supformin, a metformin dimer, disrupts the plasticity of human immune cells, dendritic cells (DCs) and macrophages. Thus, opening avenues for the development of innovative treatments (26). Given its considerable potential, metformin as an immunomodulator is increasingly featured in scientific publications. However, an integration of data and visual analysis is essential to better understand the developments in this domain.

This bibliometric study focuses on the direct relationship between metformin and its immunomodulatory effects. Bibliometric analysis is widely used to quantitatively evaluate published studies and predict future trends. This type of research is based on existing literature involving different countries, institutions, authors, journals, and keywords. It uses mathematical and statistical tools to quantify and predict the status quo of scientific research to objectively evaluate the knowledge framework and identify research hotspots. Recently, the number of research publications in this field has increased rapidly. Nonetheless, bibliometric analyses of metformin as an immunomodulator remain limited. This study evaluated published research on metformin’s role as an immunomodulator over the past decade and aimed to provide new insights for academic dynamics, drug development, and disease treatment, offering a broad perspective and roadmap for future research.

## 2 Materials and methods

### 2.1 Search strategy

This study collected research on metformin and immune regulation published from January 1, 2013, to October 1, 2024, from the WOS core collection database. Data collection was completed on October 8, 2024. The search utilized the following keywords ((TS=(Metformin OR Dimethylbiguanidine OR Dimethylguanylguanidine OR Glucophage)) AND (TS=(Immunity OR Immunization OR Immunological OR Immune))).

### 2.2 Data collection

**Figure 1:**
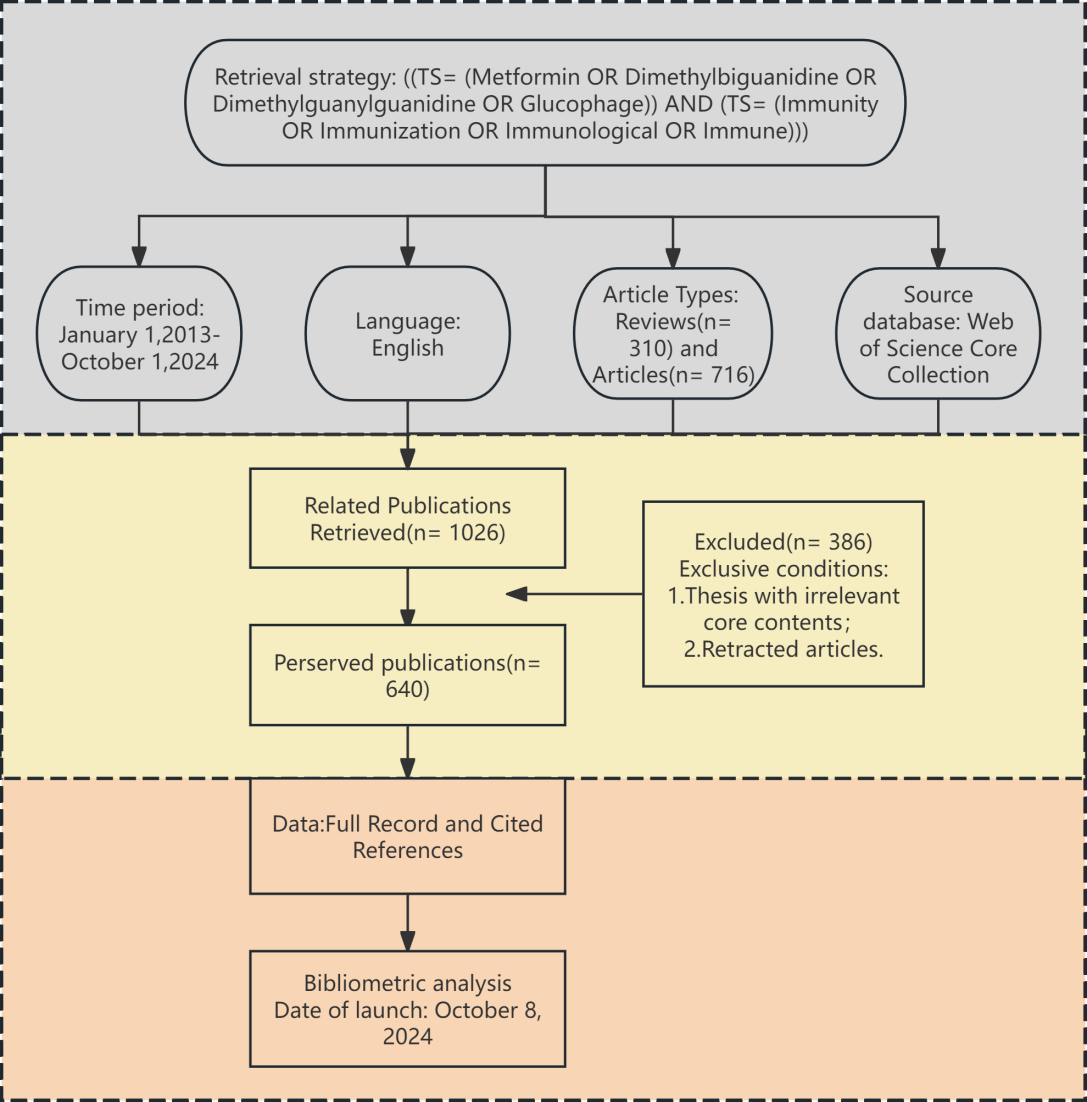
Flow diagram presenting the selection process for studies on metformin as an immunomodulator. This diagram depicts the screening of 640 publications published between January 2013 and October 2024.

To ensure the authenticity and reliability of the study, two researchers collaborated closely in retrieving and thoroughly screening the data. By excluding irrelevant literature individually, a total of 640 papers were analyzed. The detailed data screening process is illustrated in **Figure 1**. All retrieved literature that met the established criteria were used in the bibliometric analysis. The complete records and reference lists were extracted and saved as “txt” files, which served as the data source for the analysis.

### 2.3 Data analysis and visualization

We used CiteSpace (version 6.4.R1), VOSviewer (version 1.6.20), and Microsoft Excel 365 for analyzing and presenting data. VOSviewer (27) was used to extract detailed information of countries, institutions, journals, authors, citations, and keywords, as well as to construct a network visualization map. Each node in VOSviewer represented an entity, and its size was related to its weight. The thickness of the connection between the nodes was closely related to the strength of cooperation, co-citation and co-occurrence. We used CiteSpace (version 6.4.R1) (28) to calculate the burst of references, timeline of keywords, and burst of keywords to visualize the data. Microsoft Excel 365 was utilized to visualize the annual count of literature, countries, institutions, authors, journals, and the most cited local references.

## 3 Results

### 3.1 Quantitative analysis of publications output

**Figure 2:**
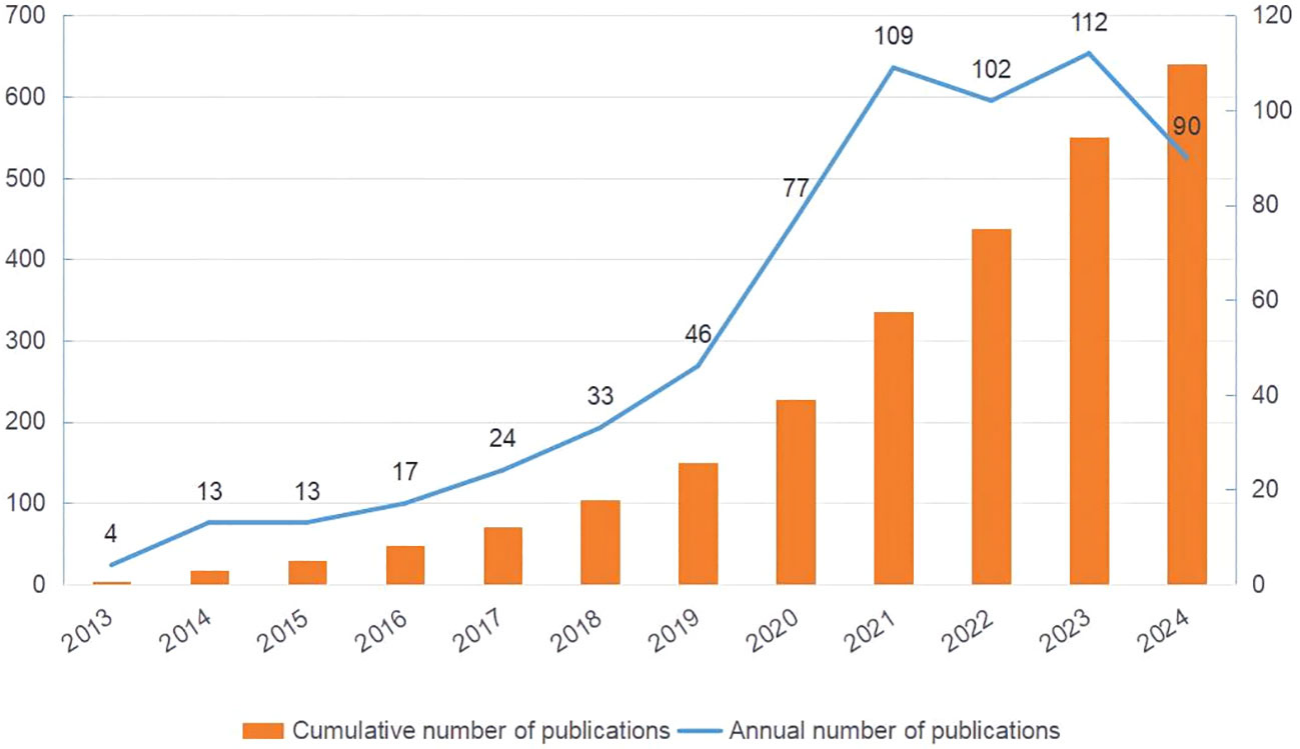
Publication trends of metformin as an immunomodulator. The orange bar chart shows the cumulative number of publications, and the blue line chart represents the annual number of publications. The horizontal axis marks the years, the left vertical axis indicates the cumulative publication count, and the right vertical axis denotes the annual publication count.

This study retrieved and reviewed effective literature from 2013 to 2024 in the WOS core database, screening 640 papers from 4,354 authors across 77 countries and 1,246 institutions. **Figure 2** demonstrates a steady increase in the number of articles related to metformin and immune regulation over time. This trend indicated that researchers paid little attention to the immunomodulatory mechanism of metformin between 2013 and 2016, with fewer than 20 articles published annually. As the potential therapeutic value of metformin’s immune regulation was better understood, there was a rapid increase on its research over the following eight years. By 2021, the annual number of publications exceeded 100, which was nearly ten times the previous number. This demonstrated that metformin as an immunomodulator has garnered significant interest from researchers.

### 3.2 Country and institutional analysis

#### 3.2.1 Inter-country research cooperation network

**Figure 3:**
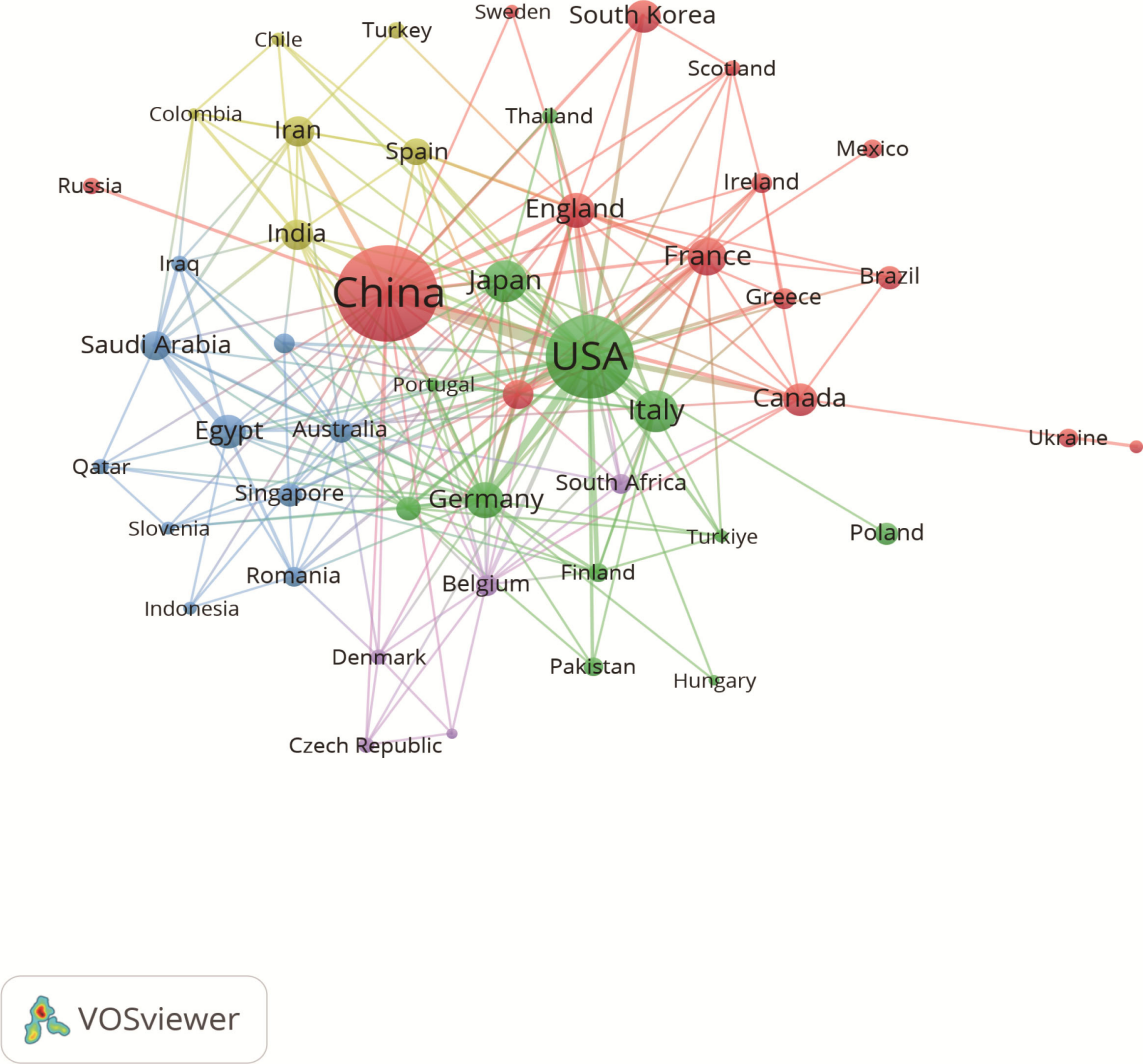
Associations between countries with more than two articles. Each circle represents a node, with larger nodes indicating greater influence of a country. The thickness of the lines denotes the strength of collaboration between two countries.

A total of 77 countries are conducting research on the immunomodulating properties of metformin. **Table 1** highlights the top 10 countries based on published articles, with China (n=217) and United States of America (USA) (n=158) accounting for 58.60% of the total number of published articles. This highlights the dominance of these two countries in this field. Notably, although USA has fewer publications than China, the average citation per article (47.95 times) and h-index (47) are higher, indicating greater research quality and influence. Additionally, the size of each node in **Figure 3** and the thickness of the connections between adjacent nodes reflect the degree of cooperation between countries. For example, in addition to its deep cooperation with China, USA also has positive cooperative relationships with Germany, Italy, Canada, Japan, and France.

**Table 1:** Top 10 countries in the field of Metformin as an immunomodulator.

Rank	Country	Documents	Percent (%)	Total citations	Average citations	H-index
1	China	217	33.91	5942	27.38	42
2	USA	158	24.69	7576	47.95	47
3	Italy	36	5.63	1182	32.83	22
4	Japan	35	5.47	1349	38.54	19
5	France	28	4.38	1171	41.82	20
6	Germany	26	4.06	622	23.92	18
7	England	24	3.75	1179	49.13	21
8	Egypt	22	3.44	198	9.00	10
9	Canada	21	3.28	745	35.48	19
10	South Korea	20	3.13	2246	112.30	17

#### 3.2.2 Inter-agency cooperation network

**Figure 4:**
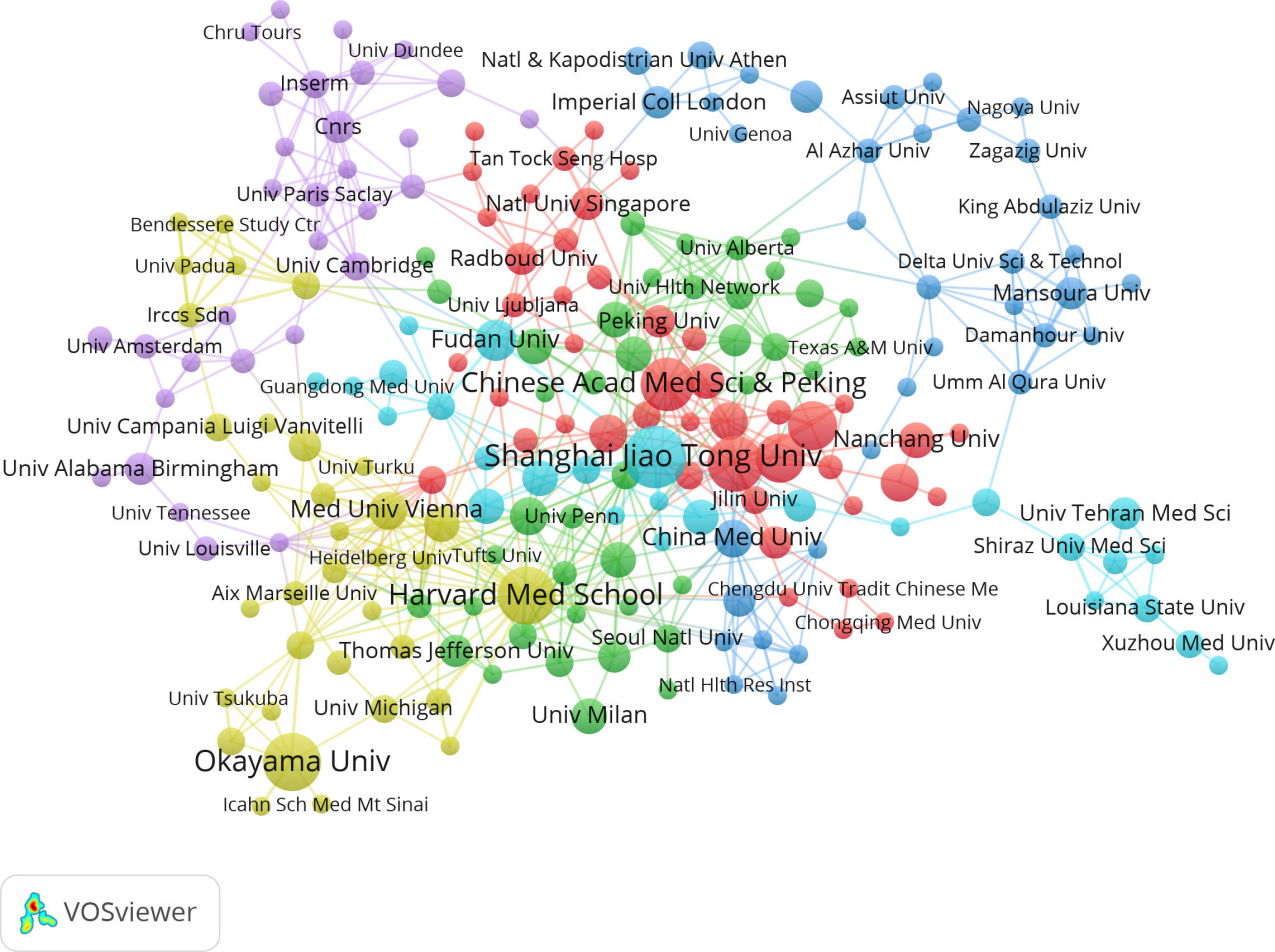
Correlations between institutions with more than two articles. In the network, larger nodes indicate greater importance of an institution and thicker lines show a higher frequency of collaborations between institutions.

The studies were performed 1,246 institutions. **Table 2** shows the top 14 institutions with more than 7 related publications, including 10 from China, 2 from USA, and 1 from Japan and Austria. Shanghai Jiao Tong Univ (n=17) published the most papers, followed by Okayama Univ (n=15), and Harvard Med School (n=15). Notably, China Med Univ (average citation rate 82.14) and Northwestern Univ (average citation rate 72.14) achieved higher average citation rates. Additionally, the visual map in **Figure 4** shows a close cluster effect among different institutions. Specifically, Harvard Med School (Total link strength [TLS]=45), Chinese Acad Sci [TLS=26] exhibit extensive collaborations with other institutions.

**Table 2:** Top 14 institutions in the field of Metformin as an immunomodulator.

Rank	Institution	Documents	Country	Total citations	Average citations
1	Shanghai Jiao Tong Univ	17	China	1017	59.82
2	Harvard Med School	15	USA	830	55.33
3	Okayama Univ	15	Japan	871	58.07
4	Chinese Acad Sci	14	China	274	19.57
5	Chinese Acad Med Sci & Peking Union Med Coll	13	China	304	23.38
6	Cent South Univ	11	China	230	20.91
7	Huazhong Univ Sci & Technol	11	China	311	28.27
8	Fudan Univ	8	China	200	25.00
9	Med Univ Vienna	7	Austria	195	27.86
10	Northwestern Univ	7	USA	505	72.14
11	China Med Univ	7	China	575	82.14
12	Nanjing Univ	7	China	433	61.86
13	Sun Yat Sen Univ	7	China	215	30.71
14	Zhejiang Univ	7	China	82	11.71

#### 3.2.3 Cooperation network among authors

**Figure 5:**
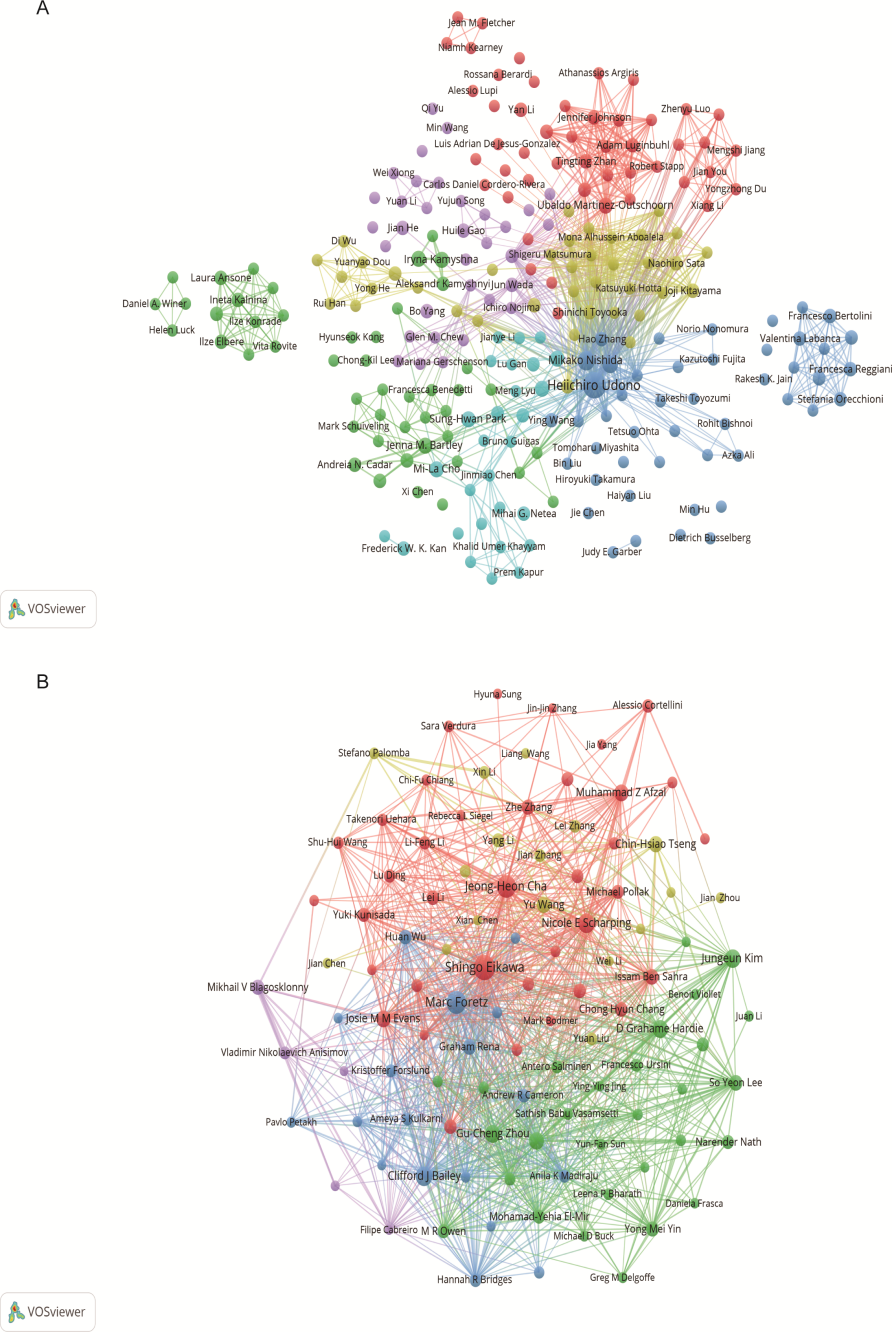
Author visualization in metformin as an immunomodulator. **(A)** Cited Author Map: This map includes authors cited in at least two studies. Larger nodes indicate higher citation frequencies, and thicker lines denote closer collaborations in the field of metformin and immunology research. **(B)** Co-cited Author Co-occurrence Map: This map features authors with more than 20 co-citations. Node size and line thickness reflect the frequency of co-citations and the strength of collaborations, respectively. Node colors represent distinct co-cited author collaboration networks.

Through screening, a total of 4,353 authors and 28,209 co-cited authors were identified. **Table 3** gathers the top 10 most prolific and most frequently co-cited authors. Heiichiro Udono (n=13) emerged as the most prolific author, followed by Mikako Nishida (n=6) and Shingo Eikawa (n=5). Notably, Shingo Eikawa stood out with the highest average citation count of 129.6, highlighting substantial academic influence. **Figure 5A** illustrates a close collaborative relationships among these cited authors. Furthermore, **Figure 5B** showcases authors with more than 20 co-citations, with the top three being Shingo Eikawa (113 citations), Marc Foretz (97 citations), and Jeong-Heon Cha (91 citations), indicating the important contributions of these authors in these relevant research fields.

**Table 3:** Top 10 most prolific authors and the 10 most frequently co-cited authors with the highest citation counts.

Author	Documents	Citations	Average citations	Co-cited author	Citations
Heiichiro Udono	13	843	64.85	Shingo Eikawa	113
Mikako Nishida	6	589	98.17	Marc Foretz	97
Shingo Eikawa	5	648	129.60	Jeong-Heon Cha	91
Jenna M. Bartley	4	38	9.50	Nicole E Scharping	70
Mi-La Cho	4	180	45.00	Clifford J Bailey	68
Iryna Kamyshna	4	27	6.75	Jungeun Kim	63
Xin Li	4	236	59.00	D Grahame Hardie	59
Yang Li	4	207	51.75	Gu-Cheng Zhou	57
Ubaldo Martinez-Outschoorn	4	92	23.00	Erika L Pearce	56
Sung-Hwan Park	4	182	45.50	Josie M M Evans	52

#### 3.2.4 Cooperation network among journals

**Figure 6:**
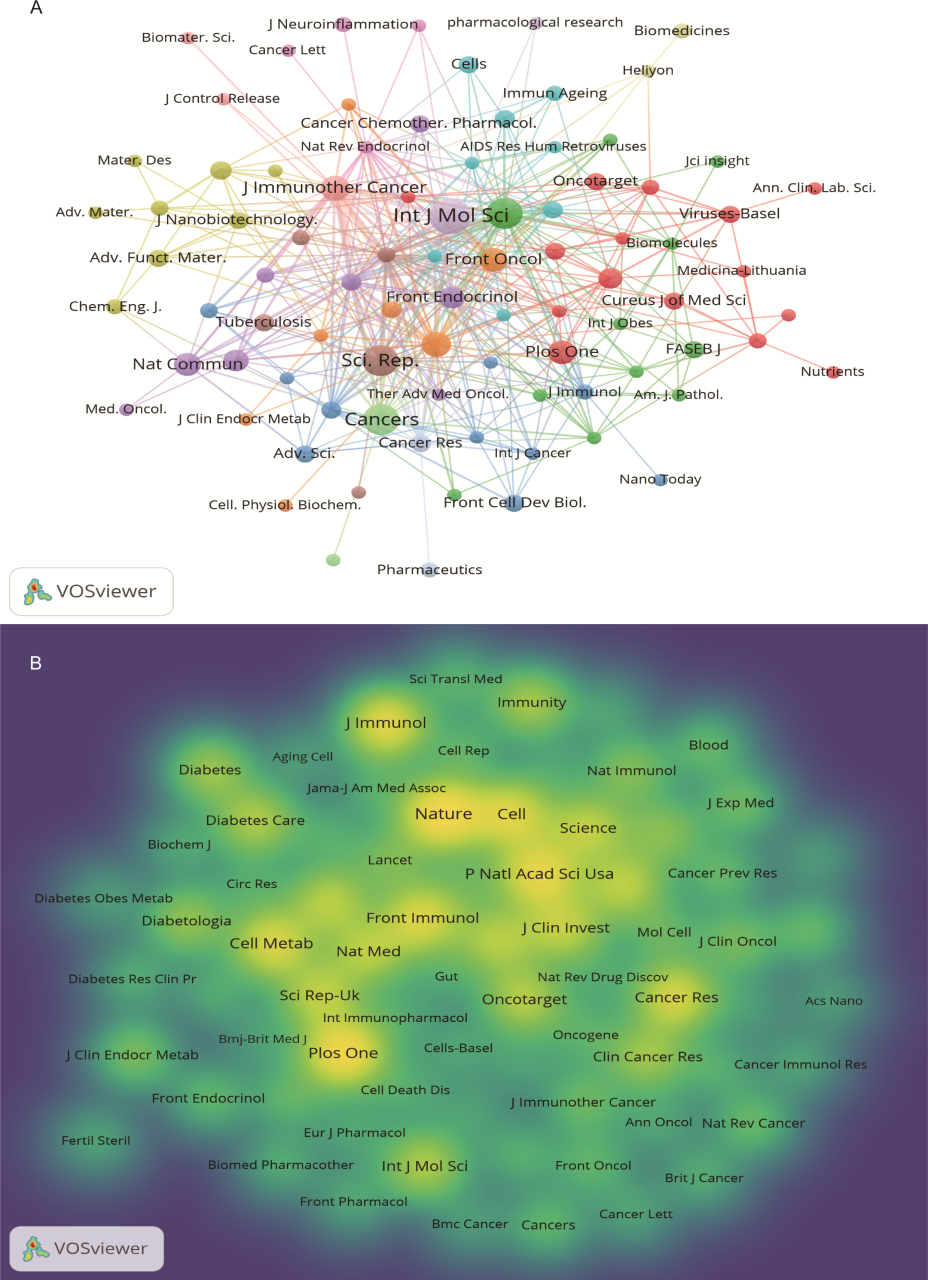
Journal visualization of metformin as an immunomodulator. **(A)** Cited Journal Co-occurrence Network: Node size represents the influence of journals within the network. Nodes of the same color indicate journal groups with close research connections. **(B)** Co-cited Journal Density Map: Brightly colored and high-density areas represent journals with high citation frequency, indicating the importance in the field.

The 640 articles included in this study are from 380 journals and 4,438 co-cited journals. The top 10 cited and co-cited journals that published metformin and immunotherapy-related papers are listed in **Table 4**. Based on the collaboration network of cited journals shown in **Figure 6A** and the 2024 *Journal Citation Report (JCR)*, *International Journal of Molecular Sciences* has published the most papers (n=22, IF=4.9), followed by *Cancers* (n=6, IF=4.5), *Frontiers in Immunology* (n=16, IF=5.7), *Scientific Reports* (n=16, IF=3.8), *International Immunopharmacology* (n=10, IF=4.8), and *Journal for Immunotherapy of Cancer* (n=10, IF=10.3). The VOSviewer co-occurrence analysis showed that *Nature* (IF=50.5), *Plos One* (IF=2.9), and *Proceedings of the National Academy of Sciences of the United States of America (PNAS)* (IF=9.4) had higher co-citation rates (**Figure 6B**).

**Table 4:** Top 10 cited journals and co-cited journals that published literatures on metformin as an immunomodulator.

Journal	Articles Counts	IF	Co-cited Journal	Cocitation	IF
*International Journal of Molecular Sciences*	22	4.9	*Nature*	829	50.5
*Cancers*	16	4.5	*Plos One*	768	2.9
*Frontiers in Immunology*	16	5.7	*Proceedings of the National Academy of Sciences of the United States of America*	664	9.4
*Scientific Reports*	16	3.8	*Journal of Immunology*	653	3.6
*International Immunopharmacology*	10	4.8	*Cell*	616	45.5
*Journal for Immunotherapy of Cancer*	10	10.3	*Cell Metabolism*	597	27.7
*Frontiers In Oncology*	9	3.5	*Frontiers in Immunology*	588	5.7
*Plos One*	9	2.9	*Cancer Research*	575	12.5
*Nature Communications*	8	14.7	*Science*	470	44.7
*Frontiers in Pharmacology*	7	5.6	*Journal of Clinical Investigation*	467	13.3

#### 3.2.5 Citation and co-citation literature network analysis

**Figure 7:**
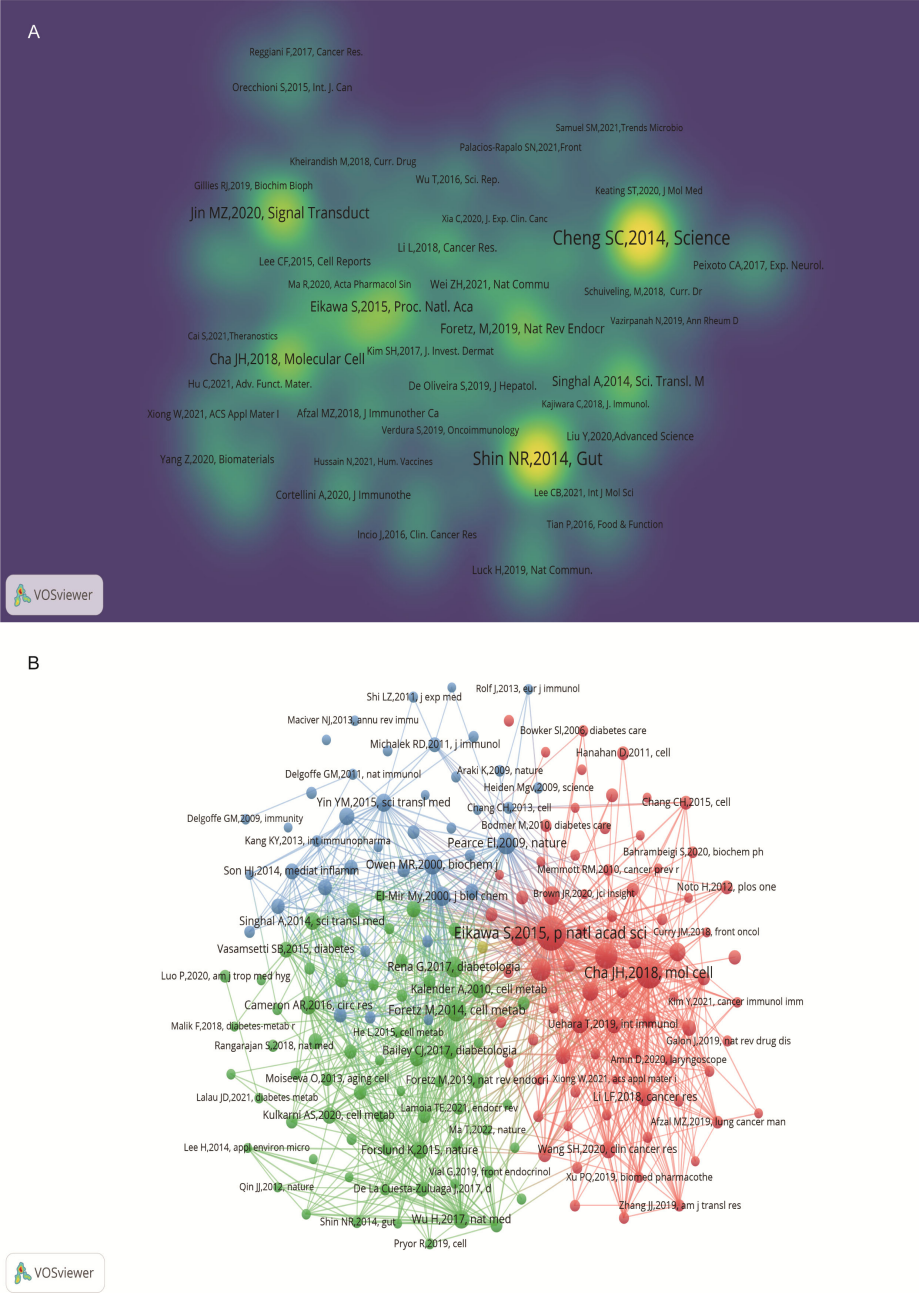
Visualization of cited references on metformin as an immunomodulator. **(A)** Cited Literature Cluster Analysis: The size of each node is proportional to the citation frequency in literature. Larger nodes indicate higher influence within the network. **(B)** Co-cited Literature Density Map: Brightly colored and concentrated areas represent literature with higher co-citation frequency, indicating greater importance in the field.

**Figure 8:**
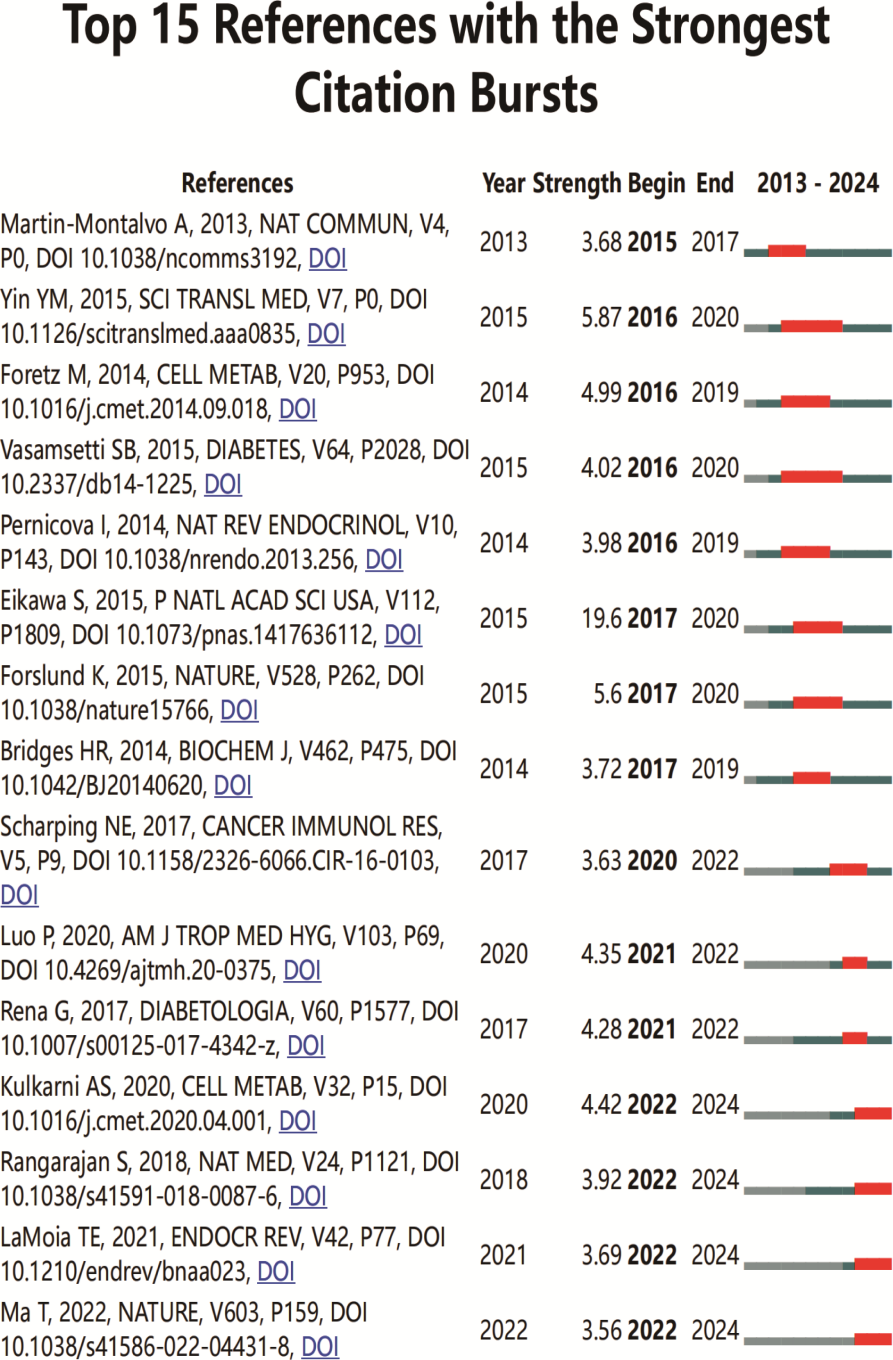
Top 15 co-cited references on metformin as an immunomodulator. Blue line segments represent the timeline from 2013 to 2024, and red markers indicate the specific period and duration of reference bursts.

An analysis of the top 10 cited and co-cited references using VOSviewer is presented in **Tables 5** and **6**, with their collaborative networks illustrated in **Figures 7A** and **B**. Among the top-cited references, the leading three are Cheng SC. et al. (2014, *Science*, 1,432 citations); Shin NR et al. (2014, *Gut*, 1,176 citations); and Jin MZ. et al. (2020, *Signal Transduction and Targeted Therapy*, 657 citations). These are followed by Cha JH et al. (2018, *Nature Immunology*, 522 citations) and Eikawa S. et al. (2015, *PNAS*, 415 citations). For co-cited references, the top three are by Eikawa S. et al. (2015, *PNAS*, 111 citations); Cha JH et al. (2018, *Molecular Cell*, 87 citations); and Scharping NE. et al. (2017, *Cancer Immunology Research*, 63 citations). **Figure 8** highlights the top 15 references with the most significant increase in citations since 2013. Notably, the study by Eikawa S. et al., published in 2015, on the anti-tumor immunotherapy effects of metformin, demonstrates the highest citation burst intensity. Moreover, many references continue to show increasing citation rates, indicating that research into the immune mechanisms of metformin remains a prominent and active area of investigation.

**Table 5:** Top 10 most cited references of Metformin as an immunomodulator.

Author	Title	Journal	Citation	Year
Cheng SC. et al.	Mtor- and hif-1α-mediated aerobic glycolysis as metabolic basis for trained immunity	*Science*	1432	2014
Shin NR. et al.	An increase in the akkermansia spp. population induced by metformin treatment improves glucose homeostasis in diet-induced obese mice	*Gut*	1176	2014
Jin MZ. et al.	The updated landscape of tumor microenvironment and drug repurposing	*Signal Transduction And Targeted Therapy*	657	2020
Cha JH. et al.	Metformin promotes antitumor immunity via endoplasmic-reticulum-associated degradation of pd-L1	*Molecular Cell*	522	2018
Eikawa S. et al.	Immune-mediated antitumor effect by type 2 diabetes drug, metformin	*PNAS*	415	2015
Foretz M. et al.	Understanding the glucoregulatory mechanisms of metformin in type 2 diabetes mellitus	*Nature Reviews Endocrinology*	376	2019
Scharping NE. et al.	Efficacy of pd-1 blockade is potentiated by metformin-induced reduction of tumor hypoxia	*Cancer Immunology Research*	373	2017
Singhal A. et al.	Metformin as adjunct antituberculosis therapy	*Science Translational Medicine*	360	2014
Li L. et al.	Metformin-induced reduction of cd39 and cd73 blocks myeloid-derived suppressor cell activity in patients with ovarian cancer	*Cancer Research*	204	2018
Song M. et al.	Environmental factors, gut microbiota, and colorectal cancer prevention	*Clinical Gastroenterology And Hepatology*	194	2019

**Table 6:** Top 10 most co-cited references concerning Metformin as an immunomodulator.

Author	Title	Journal	Citation	Year
Eikawa S. et al.	Immune-mediated antitumor effect by type 2 diabetes drug, metformin	*PNAS*	111	2015
Cha JH. et al.	Metformin promotes antitumor immunity via endoplasmic-reticulum-associated degradation of PD-L1	*Molecular Cell*	87	2018
Scharping NE. et al.	Efficacy of PD-1 blockade is potentiated by metformin-induced reduction of tumor hypoxia	*Cancer Immunology Research*	63	2017
Evans JMM. et al.	Metformin and reduced risk of cancer in diabetic patients	*BMJ (British Medical Journal)*	48	2005
Foretz M. et al.	Metformin: from mechanisms of action to therapies	*Cell Metabolism*	45	2014
Pearce EL. et al.	Enhancing CD8 T-cell memory by modulating fatty acid metabolism	*Nature*	45	2009
Rena G. et al.	The mechanisms of action of metformin	*Diabetologia*	40	2017
Wheaton WW. et al.	Metformin inhibits mitochondrial complex I of cancer cells to reduce tumorigenesis	*eLife*	38	2014
Owen MR. et al.	Evidence that metformin exerts its anti-diabetic effects through inhibition of complex 1 of the mitochondrial respiratory chain	*Biochemical Journal*	37	2000
Kunisada Y. et al.	Attenuation of CD4+CD25+ Regulatory T Cells in the Tumor Microenvironment by Metformin, a Type 2 Diabetes Drug	*EBioMedicine*	36	2017

### 3.3 Keywords and hotspots

**Figure 9:**
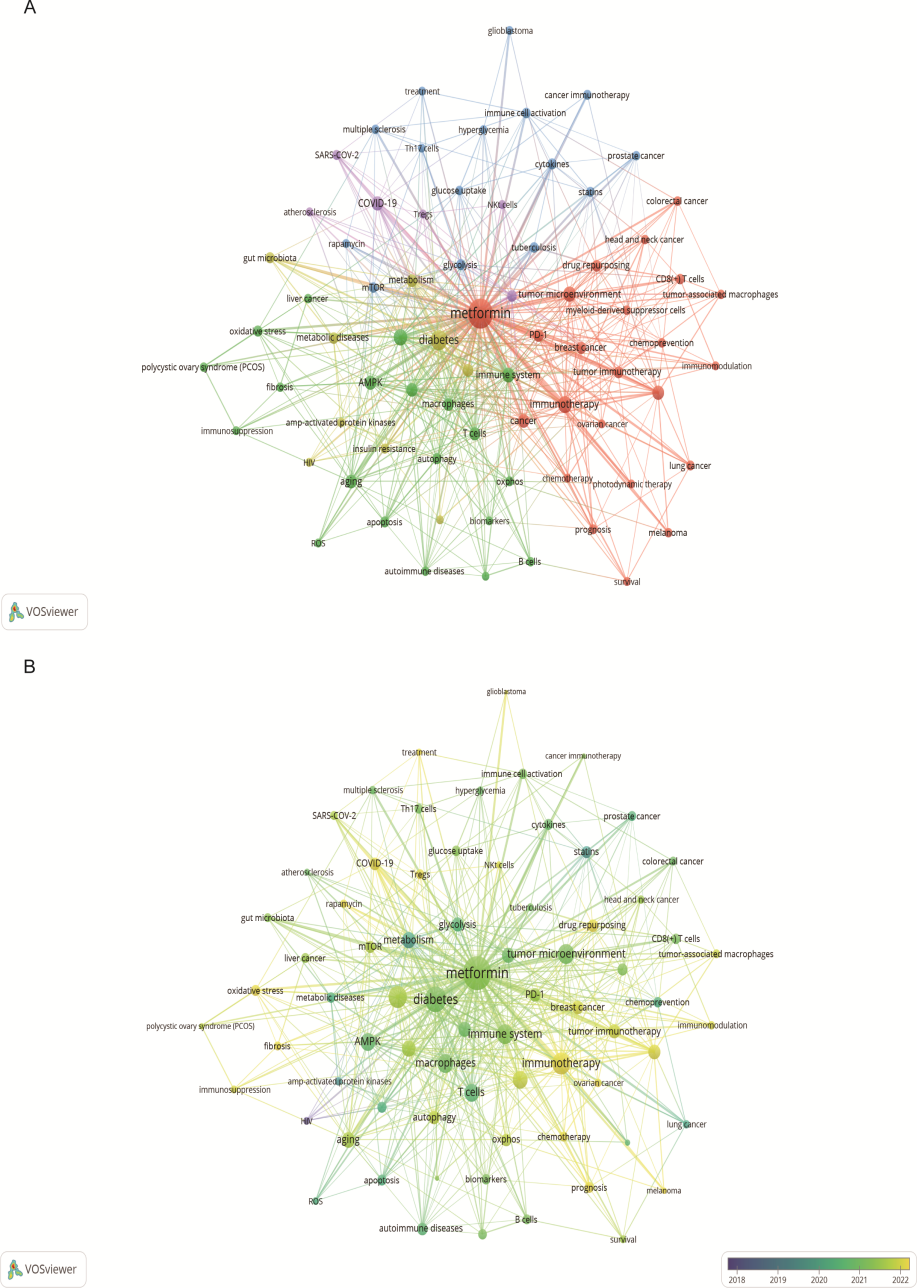
Keyword co-occurrence and annual analysis related to metformin as an immunomodulator. **(A)** Keyword Co-occurrence Network: Node size corresponds to the frequency of keyword occurrences, and the lines between nodes indicate the strength of the association. Larger nodes denote higher frequencies, and thicker lines represent stronger relationships between keywords. **(B)** Annual Analysis of Keyword Occurrence: Each node represents a keyword, with its color indicating the average year of occurrence. This visualization shows the research intensity of keywords over specific periods, highlighting the research focus in different time frames.

**Figure 10:**
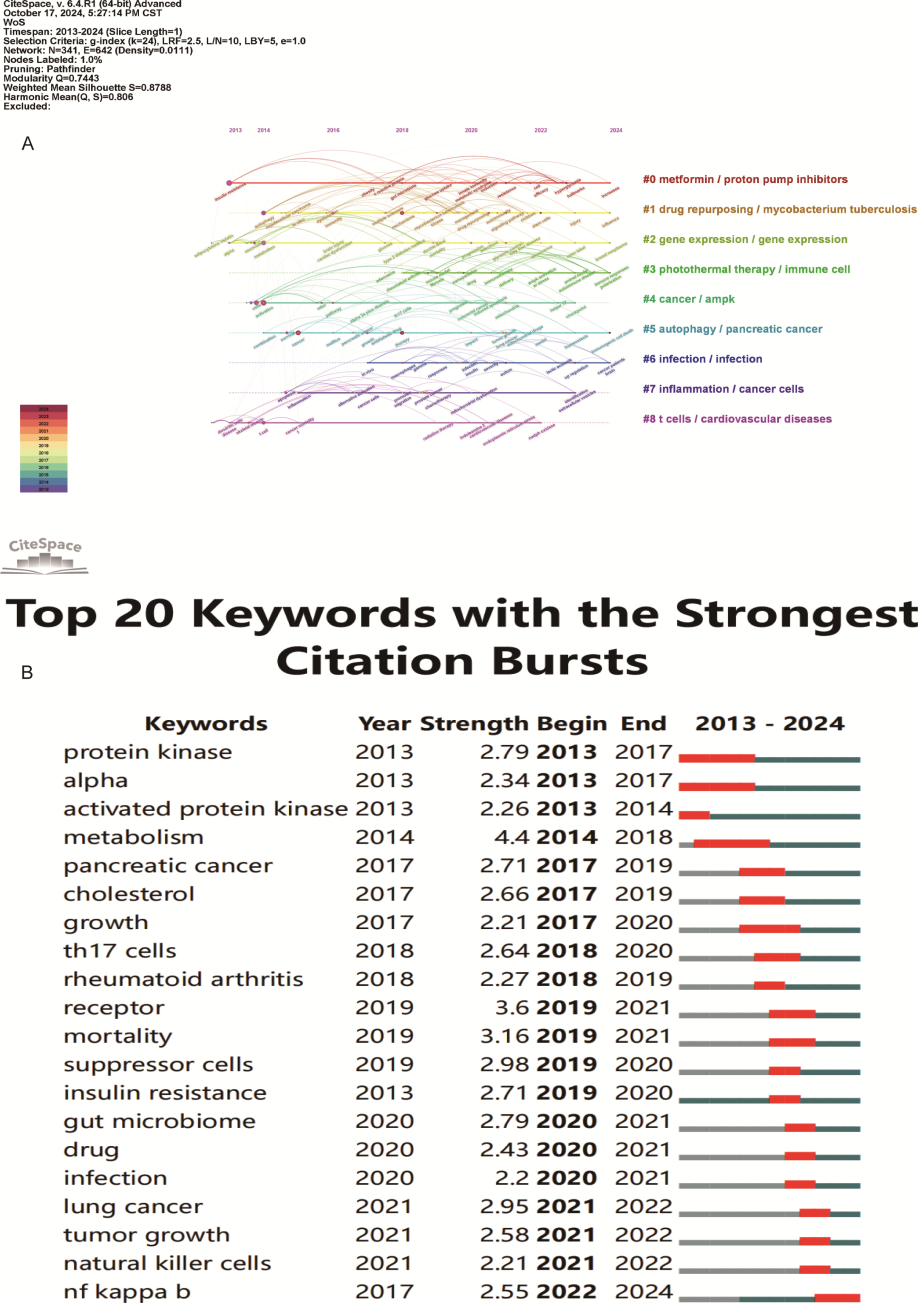
Keyword timeline and burst word analysis of metformin’s role as an Immunomodulator. **(A)** Keyword Cluster Timeline: Keywords within the same cluster are aligned on the same horizontal line. This visualization shows the number of keywords in each cluster and the time span, indicating the temporal evolution of various research directions. **(B)** Top 20 Keyword Burst Analysis: Red line segments indicate the start and end of a keyword burst. The “strength” metric represents the intensity of research on a keyword during a specific period, with higher values indicating greater research focus and attention.

Keyword analysis showed the dynamic progress of metformin’s immune regulation. Using the “author keywords” mode in VOSviewer, a total of 1,615 keywords were obtained. After merging synonyms, 1,537 keywords were analyzed. **Table 7** lists the top 20 keywords with the highest frequency, and **Figure 9A** displays the co-occurring keyword analysis on metformin and immune regulation research showing the evolution of research focus from 2013 to 2024. The top three core keywords were “metformin” (293 occurrences), “diabetes” (78 occurrences), and “immunotherapy” (44 occurrences), followed by “inflammation” (37 occurrences) and “tumor microenvironment” (30 occurrences). These topics have consistently attracted significant interest. Using the density view (**Figure 9B**) and timeline diagram (**Figure 10A**) of time evolution, it was observed that since the year 2013, the focus on metformin’s immune regulatory properties gradually shifted from metabolic diseases and insulin resistance to tumor immunity, autoimmune diseases, COVID-19, and aging. The mechanisms broadly involve oxidative stress, inflammation, intestinal flora, immunosuppressive checkpoints, the tumor immune microenvironment, and oxidative glycolysis.

**Table 7:** Top 20 keywords related to metformin as an immunomodulator.

Rank	Keyword	Occurrences	Rank	Keyword	Occurrences
1	metformin	293	11	cancer	21
2	diabetes	78	12	mitochondrial dysfunction	21
3	immunotherapy	44	13	T cells	19
4	inflammation	37	14	macrophages	17
5	tumor microenvironment	30	15	metabolism	17
6	immune system	29	16	obesity	16
7	AMPK	25	17	PD-1	16
8	COVID-19	24	18	breast cancer	15
9	immune checkpoint inhibitors	24	19	immunometabolism	13
10	aging	21	20	tumor immunotherapy	13

From the keyword bursts, the top 20 keywords with the highest burst intensity are shown in **Figure 10B**. Over the past decade, the focus of metformin immunotherapy has shifted from metabolic and diabetic diseases to the treatment outcomes and mechanisms of tumors. Currently, research is predominantly focused on metformin as an immunomodulator in tumor immunity; however, other mechanisms such as inflammation and intestinal flora are also being explored.

## 4 Discussion

### 4.1 General information

This study conducted a visual analysis on data from 640 publications obtained from WOS, using CiteSpace and VOSviewer. The results depict trends in annual publication volume, geographical and institutional cooperation, journal distribution, author co-occurrence, literature cross-citation, and research hotspots. In 2013, this field was in its infancy; however, the academic output increased significantly from 2017 to 2023, with a publication peak observed in 2020. It is expected that the number of articles will continue to grow in 2024 and beyond suggesting of increased interest and focus in this field.

In modern research, the immune response is a crucial factor for understanding the therapeutic mechanism of metformin. Data from various countries and academic institutions showed that most research with high publication volume and h-index has been published from the USA, which has a well-established cooperation network with other countries. Strengthening overall communication would benefit academic development in this field. Analysis of institutional cooperation showed that Chinese institutions dominate research related to metformin immunomodulatory therapy. Domestic institutions frequently cooperate and exhibit regional collection characteristics. The top 3 institutions according to literatures are Shanghai Jiao Tong Univ (17 papers), Okayama Univ (15 papers), and Harvard Med School (15 papers). Regarding journal distribution, the *International Journal of Molecular Sciences* leads in publication frequency with 22 papers, highlighting its exemplary role in this area of research. The hierarchical structure of journals showed that *Cancers, Frontiers in Immunology*, and *Scientific Reports* were the key publishers of this research. Additionally, *Nature* (co-citation = 829) was the most frequently co-cited journal, with the highest impact factor of 50.5. Heiichiro Udono from Okayama University in Japan was a prolific author in this domain (n=13). He collaborated with Shingo Eikawa on studies investigating the enhanced effect of cancer immunotherapy through metabolic intervention with metformin, and has published several highly cited articles (29, 30). These research results provided new perspectives and potential therapeutic strategies for the application of metformin in cancer immunotherapy, deepening the understanding of its immunoregulatory mechanisms (31–33). The top 10 references mainly focus on tumor immunity, tumor hypoxia, the tumor microenvironment, immune checkpoint inhibition, anti-inflammatory effects, tuberculosis, polycystic ovary syndrome, gut microbiota, and the endoplasmic reticulum pathway. This highlights the immunomodulatory role of metformin in various diseases, etiologies, and pathological mechanisms. Its wide application offers significant guidance for both basic and clinical research.

### 4.2 Hotspots and frontiers

#### 4.2.1 Metformin-mediated tumor immunity

The study of high-frequency keywords can clarify the hot spot distribution and trajectory on research elated to metformin and host immunity research over the recent years. Previously, researchers have demonstrated promising anticancer effects of metformin on prostate, breast, lung, liver, colon, ovarian, head and neck, glioblastoma cancers (34). Metformin is a highly effective and promising anti-tumor inhibitor. Targeted tumor immunity is an attractive research topic. As shown in the **Figure 9A**, keyword clusters such as ″tumor immunotherapy″, ″tumor microenvironment″, ″immune checkpoint inhibitors″, ″immunotherapy″, ″drug repurposing″, ″tumor-associated macrophages″, ″CD8(+) T cell″, ″lung cancer″, ″breast cancer″, ″photodynamic therapy″, ″chemoprevention″, and ″survival″ highlight the prominence of metformin’s immune-mediated effects in tumor research. These indicate that the immune-mediated role of metformin in tumor diseases is a current topic of interest. Its detailed mechanism of action involves many aspects of tumor pathogenesis, such as the tumor immune microenvironment, immune checkpoint inhibition, and the application of metformin derivatives.

Firstly, metformin significantly influences the regulation of the tumor immune microenvironment, covering a variety of immune cells and their interactions, inhibiting tumor metastasis, progression, and angiogenesis. It enhances the anti-tumor immune function of CD8+ T cells by regulating glucose metabolism, activating fatty acid oxidation, and targeting oxidative phosphorylation (35). Metformin can promote the maturation of dendritic cells by depressing mitochondrial complex I, enhance the co-stimulatory ability of CD4+ T cells, and further stimulate the immune response (36). Through the p38 mitogen-activated protein kinase pathway, metformin enhances the anti-tumor activity of natural killer cells and increases their toxicity to cancer cells (37). It also inhibits tumor infiltration of Treg and myeloid-derived suppressor cells (38), stimulates the proliferation of natural killer T and T cells (14), and delays cancer growth. Studies have shown that metformin regulates macrophage function, increases tumor-infiltrating T cells, and significantly heightened the anti-tumor effect of cancer vaccines (39, 40). When combined with antitumor drugs such as pazopanib and cyclophosphamide, metformin improves the immune microenvironment. This could be achieved by inhibiting phosphorylated protein kinase B/nuclear factor kappa-light-chain-enhancer of activated B cell/signal transducer and activator of transcription 3 (STAT3p-Akt/NF-κB/STAT3)/PD-L1 pathway (41, 42). In addition, metformin causes immunogenic death of ovarian cancer cells and inhibits tumor growth by activating AMPK (43). It improves prognosis and prolongs survival in patients with pancreatic and breast cancer by reducing the abundance of M2 macrophages and increasing tumor-infiltrating lymphocytes (TIL) (44, 45). In summary, metformin shows a wide range of anticancer potential by modulating anti-tumor immune cells, reducing immunosuppression, and significantly improving the tumor immune microenvironment.

Secondly, immune checkpoint molecules, such as PD-L1 and cytotoxic T-lymphocyte associated protein 4(CTLA4), have been extensively investigated as a cancer therapy, with this immunotherapy notably enhancing the survival rates of patients with certain types of cancer (46). Metformin, as a small molecule immune checkpoint inhibitor, decreases PD-L1 expression on tumor cell surfaces and boosts the cytotoxic T cells ability to kill (20). By activating AMPK, metformin phosphorylates PD-L1, induces endoplasmic reticulum stress, and enhances anti-tumor immunity (47). Concurrently, metformin restrains the expression of mechanistic target of rapamycin (mTOR) signaling and hypoxia-inducible factor 1-alpha (HIF-1α), decreases oxygen consumption, strengthens the efficacy of PD-1 blockade therapy, and diminishes immune escape (48, 49). In addition, metformin combined with anti-PD-1 therapy promotes the normalization of tumor blood vessels, increases the expression of vascular endothelial cadherin (VE-cadherin) and vascular cell adhesion molecule 1(VCAM-1), and infiltration of CD8+ T cells, and slows tumor growth (32). Studies have shown that metformin significantly prolonged overall survival (OS) and progression-free survival (PFS) in patients with non-small cell lung cancer (NSCLC) treated with immune checkpoint inhibitors (50). This combination therapy not only enhances the effectiveness of immunotherapy but enhances the prognosis of cancer patients.

Further, reuse of metformin has gained momentum. Due to its clear anti-cancer effect, good safety, and tolerance, but low drug concentration, researchers have developed a variety of metformin derivatives to enhance its efficacy in anti-tumor immunotherapy. The platinum-metformin conjugate promotes the degradation of PD-L1 through the AMPK/transcription factor EB (TFEB) pathway and enhances the anti-tumor immune effect in NSCLC (51). Researchers have further enhanced the therapeutic effects on cold tumors and drug-resistant tumors by using nanotechnology delivery systems combined with photodynamic therapy, radiotherapy, and immune checkpoint blockade therapy (52–54). For example, MET-HMME/CAT-HMME@Nlip (55) and Mn-MSN@Met-M NPs (56) improves tumor hypoxia, activates the stimulator of interferon genes (STING) pathway, and improves efficacy of PD-1 inhibitors. Tpp-Met@MnO2@Alb nano-adjuvant enhances the sensitivity of tumors to radiotherapy by regulating PD-L1 and transforming growth factor beta 1(TGF-β1) and provides a long-term immune memory effect (52). Nanogels (D@HPMNG) (57) significantly inhibits the growth and recurrence of melanoma by reprogramming tumor-associated macrophages (58), whereas HMMDN-Met@PM enhances tumor inhibition by promoting the transformation of M2 to M1 (59). Through these mechanisms, metformin derivatives not only improve the tumor microenvironment but also inhibit tumor growth and metastasis, providing a long-term anti-tumor immune effect.

In summary, the interplay between metformin and the immune response is a complex and dynamic research area with exciting implications. These results might have important applications in controlling cancer progression and prognosis, opening avenues for the development of innovative treatments.

#### 4.2.2 Application of metformin immunotherapy in other diseases

In addition to anti-tumor immunity, metformin has a broad application in several diseases because of its ability to regulate the immune system. Keywords clusters such as ″diabetes″, ″metabolism″, ″autoimmune diseases″, ″polycystic ovary syndrome″, ″multiple sclerosis″, ″SARS-COV-2″, ″aging″, ″obesity″, etc., are all recent research topics. Metformin improves type 1 diabetes (T1DM), autoimmune diseases, and metabolic syndrome by activating AMPK pathway, inhibiting pro-inflammatory Th1 and Th17 cells, and promoting Tregs development (60, 61). Metformin has been shown to regulate differentiation of immune complexes and B cells and reduce the inflammatory response of diseases such as systemic lupus erythematosus (62). It also improves Sjogren’s syndrome by restoring Ca2+ signaling and inhibiting ERS (63), and enhances immune recognition and lowers viral replication in human immunodeficiency virus type 1(HIV-1) infection (64) and Helicobacter pylori infection. Metformin exhibits anti-inflammatory and antiviral effects in COVID-19 infections likely improving immune response and prognosis (65). Surprisingly, it can also promote osteogenic differentiation of bone marrow mesenchymal stem cells by regulating the macrophage phenotype (66) and enhancing innate immunity in tuberculosis (67, 68). In addition, metformin exhibits anti-aging and anti-fibrosis effects by reducing the number of senescent T cells and improving T cell function (69) and exhibits immunomodulatory effects in diseases such as polycystic ovary syndrome, pregnancy, and liver disease (70).

#### 4.2.3 Research hotspots on the basic mechanism of metformin immunotherapy

A clustered analysis of existing keywords showed that metformin regulated direct immune cells and also regulated immunity through other pathways involving ″fibrosis″, ″oxidative stress″, ″inflammation″, ″apoptosis″, ″ROS″, ″mTOR″, ″AMPK″, ″mitochondrial dysfunction″, ″gut microbiota″, and ″OXPHOS″, and ″mTOR″ signaling (71, 72). In addition, metformin can inhibit the differentiation of pro-inflammatory macrophages (M1) and reduce the production of reactive oxygen species (ROS) showing a strong anti-inflammatory effect (73, 74). By regulating reactive oxygen/nitrogen levels and immune metabolism, metformin may help prevent age-related diseases and provide a new direction for clinical treatment (75). It improves diabetic complications and T cell function in patients with COVID-19 by reprogramming dendritic cell metabolism, inducing tolerance, and reducing pro-inflammatory factors such as IL-2 and tumor necrosis factor alpha (76). Metformin also reduces inflammation caused by obesity and insulin resistance by regulating Th17/Treg balance, intestinal flora, and NF-κB signaling pathway (77, 78). Furthermore, it improves efficacy of anti-PD-L1 antibodies by affecting the intestinal microbiota, promotes anti-tumor immunity (79), and shows antiviral potential for COVID-19 treatment (80). In short, the relationship between anti-inflammatory, intestinal flora, and metformin immune regulation is an emerging research field. Although some studies have reported on alterations in the gut microbiome after the use of metformin (78), the exact mechanism by which metformin regulates the gut microbiome and the impact of these changes on the efficacy of metformin immunotherapy is not known. Further research is required to understand these effects.

### 4.3 Advantages and limitations

This study for the first time systematically used VOSviewer and CiteSpace to analyze the bibliometrics of research related to metformin and immune regulation, providing an overview of the cooperation network and research trends. However, there are some limitations to this study. In this study, the data was sourced from only the core collection of WOS, excluding other databases and non-English publications. Further, reference delays and database updates might have also affected the accuracy of trend analysis. Nevertheless, by combining published literature, this study offered valuable insights into the clinical application of metformin as an immunomodulator. Although current *in vivo* and *in vitro* studies have identified important mechanisms underlying the inhibitory effect of metformin on cancer immunity and other diseases, systematic and large-center clinical research studies are warranted.

## 5 Conclusion

This study used bibliometric analysis to review the dynamics and developments in the immunomodulatory effects of metformin. With the rapid growth of research on the role of metformin and its derivatives in cancer immunotherapy, the prospects of metformin use are expanding. Notably, as an immunomodulator, metformin not only improves the tumor immune microenvironment and enhances the efficacy of immune checkpoint inhibitors but also boosts the treatment effects of radiotherapy, cold tumors, and drug-resistant tumors, along with minimizing its side effects. Beyond tumor immunity, the immunomodulatory effects of metformin in aging, tuberculosis, viral infections, and autoimmune diseases, and its potential in gut microbiota and anti-inflammatory treatments, are important for future research. Further exploration of the detailed mechanisms of metformin as an immunomodulator and its biological distribution in tissues is crucial for optimizing its clinical application.

## Data Availability

The raw data supporting the conclusions of this article will be made available by the authors, without undue reservation.
